# Straining Behavior of Mortar Reinforced by Cold Drawn Crimped and Dog-Bone-Shaped Fibers under Monotonic and Cyclic Compressions

**DOI:** 10.3390/ma14061522

**Published:** 2021-03-20

**Authors:** Ha Vinh Ho, Eunsoo Choi, Duhyeon Kim, Joowon Kang

**Affiliations:** 1 Department of Civil Engineering, Hongik University, Seoul 04066, Korea; hovinhha@mail.hongik.ac.kr (H.V.H.); nspower83@daelim.co.kr (D.K.); 2 School of Architecture, Yeungnam University, Gyeongbuk 38541, Korea; kangj@yun.ac.kr

**Keywords:** straining behavior, load type effect, SMA fiber-reinforced mortar, crimped fiber, dog-bone-shaped fiber

## Abstract

The straining behavior of the shape memory alloy (SMA) fibers-reinforced mortar was investigated in this study by the monotonic compressive and cyclic compressive tests. Two types of SMA fibers with a crimped and dog-bone shape were used due to the high pullout resistance capacity, which guaranteed that the fibers and mortar matrix were composited well. The plain mortar was mixed with two different compositions to create the higher elastic modulus mortar matrix and the lower elastic modulus mortar matrix compared with the elastic modulus of SMA fibers. The results of the experimental test indicated that the non-heated SMA fibers could control the strains in both elastic and plastic phases; in which, the crimped fiber was more effective in precracking due to the higher composite capacity while the dog-bone-shaped fiber had a higher effect in post-cracking. After heating, the dog-bone-shaped fiber slipped more than that of the crimped fiber; thus, the heated crimped fiber was more effective than the heated dog-bone-shaped fiber in controlling strains after cracking. The effect of SMA fibers on the elastic modulus depended on both the elastic modulus of mortar matrix and the property of SMA fibers. In the plastic phase, the fibers were effective on reducing the speed of damage in monotonic case. An equation using reinforcing index was suggested for damage evolution in the cyclic case.

## 1. Introduction

It is well known that mixing short and discrete fibers into the mortar randomly or uniformly increased the performance of cementitious composite not only under static, quasistatic loads but also the shock, fatigue loads. The random distributions of the fibers around coarse aggregates form as a truss structure with these fibers acting as ties and a mortar matrix as their struts. The embedded fibers bear the tension; whereas the mortar matrix carries the compressive forces and transfers the internal tensile forces to the embedded fibers. The reinforcing capacity due to the fiber mixing can arrest the opening and widening of microcracks; thus, the mortar mixing randomly dispersed fibers can enhance straining capacity both under tension and compression [1].

Strength, pullout resistance of fibers, and mortar matrix are indicated that they present the role in controlling post-cracking behavior of reinforced mortar. As steel-, shape memory alloy (SMA)-, glass-, or carbon fibers own much higher tensile strength and elastic modulus than the synthetic fibers (such as polyvinyl alcohol and polypropylene), they are investigated to be more effective in spreading the tensile forces in the mortar matrix and their transmissions to the adjoining mortar are better than the synthetic fibers. The low elastic modulus of synthetic fibers reduces their transference in tension; thus, they are less contributed to tensile/compressive behavior after cracking. This is the main reason for the less usage of synthetic fibers in cement mortar [2]. The pullout resistance associates with three bond mechanisms: adhesion, friction, and the anchoring bond. Straight fiber only has adhesive and frictional resistances, which are depended on the surface condition of fibers and mortar matrix. Different surface treatments such as acid-etched, hand sanded, and sandblasted are studied to enhance the surface condition; however, these methods are uneconomical ways [3]. Various discontinuous shapes such as hooked, twisted, and crimped shapes are produced for steel fibers and some shapes of paddled, dog-bone, crimped, L, and N are suggested for SMA fibers [4,5,6,7,8,9,10,11,12]. The shapes induce the anchoring bond beside the adhesion and friction for the fibers. The other high elastic modulus fibers such as glass and carbon fibers cannot be created as a discontinuous shape.

The SMA fibers are studied in recent decades because of the superior properties of the shape memory effect and superelasticity. The recovery stress due to the shape memory effect was the cause of crack-closing [13,14,15], prestressing [16,17], and crack-repairing [18] in the cementitious composite. The superelastic characteristic provides the self-recovering capacity under the unloading condition [19,20,21]. In reinforced mortar, the SMA fibers without heating for phase transformation play the passive fibers like steel, carbon, and glass fibers for providing only the bridging effect. However, by heating for the phase transformation, they play the role of active fibers to reduce the development of microcracks and cracks-width of macrocracks due to the recovery stress. Thus, the SMA fibers have the potential to control strain. The strain behaviors of reinforced mortar with SMA fibers in both heat treatments are not invested yet.

Some experimental studies on the pullout resistance of SMA fibers indicated that only the crimped shape and dog-bone shape are suitable for max production, and they provided reasonable pullout resistance [21]. Thus, for the widely applying in construction and ensuring composite capacity of fibers and mortar matrix, the crimped shape and the dog-bone shape are used to investigate the effect on controlling axial and lateral strains under both monotonic compressive load and cyclic compressive load.

## 2. Experimental Program

### 2.1. Material

**Figure 1:**
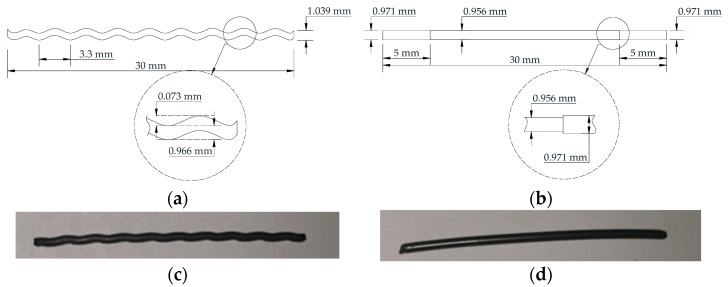
Shapes and dimensions of the fibers, (**a**) crimped fiber, (**b**) dog-bone-shaped fiber, (**c**) photo of a crimped fiber, and (**d**) photo of a dog-bone-shaped fiber.

The cold drawn Ni-Ti SMA fibers were used in this study. Firstly, the 1.0 mm diameter of Ni50.4-Ti (wt.%) SMA fibers were cold drawn with heating and cooling processes to introduce the prestrain for the fibers. Then, the crimped shape was made by crimping methods while the dog-bone shape was produced by heating the end parts. The shapes and dimensions of both fibers were presented in Figure 1.

**Figure 2:**
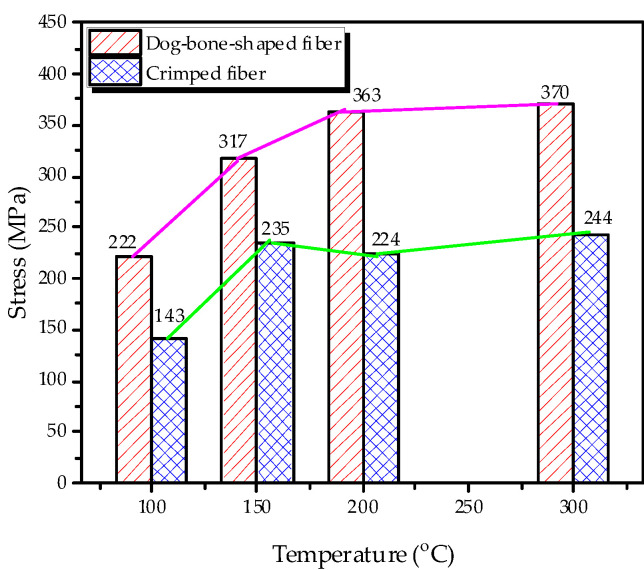
Comparison maximum recovery stress of the fibers.

The fibers in this study had a high tensile strength of about 950 MPa and an elastic modulus of 21.2 GPa. The 0.5% and 1.0% fiber contents were used for the monotonic test, while the fiber volume fraction of 1.0% and 1.5% were endowed into the cyclic compressive specimens. The maximum recovery stress of the fibers increased with increasing the heating temperature as shown in Figure 2. Meanwhile, the crimped fiber showed relatively low recovery stress for all heating temperature because of the stretching effect.

**Figure 3:**
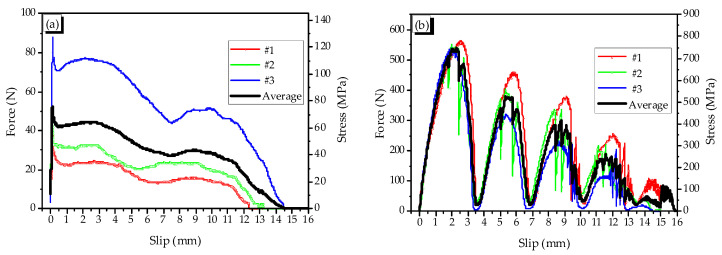

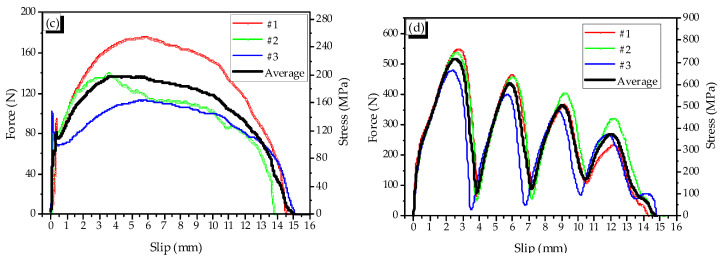
Pullout force–slip relation in non-heating and heating cases, (**a**) Non-heated dog-bone-shaped fiber, (**b**) Non-heated crimped fiber, (**c**) Heated dog-bone-shaped fiber (**d**) Heated crimped fiber.

Pullout behaviors of the fibers were also shown in Figure 3. For the dog-bone-shaped fiber, it got the maximum pullout force at the slip of about 0.1–0.3 mm, which was very small in comparison with the total slip. When being heated, the fiber’s diameter was bulged; thus, the additional frictional bond was developed due to the confining pressure induced by the bulging. The additional frictional bond and the end anchoring increased the pullout resistance resulting in that the slip at the peak pullout force moved to about 6 mm. For crimped fiber, the shape of the responsive pullout force-slip curve was a look-a-like of the wave with the wavelength that was nearly equal to the step of each indentation of the crimped fiber. The pullout force of crimped fiber was relatively high; the maximum values were about 550 N and 520 N in non-heating and heating cases, respectively. The peak of the pullout force was at the peak of the crimped part of the fiber in both cases of heating treatment.

The mortar matrix was produced with different target compressive strengths for the monotonic and cyclic tests. The plain mortar for the monotonic test using Portland cement type I, silica sand, fly ash, water, and admixture to reduce water; the ratio by weight was 1:1:0.15:0.35:0.009. The mortar for the cyclic test did not use water-reducing admixture; however, the volume fraction of fly ash was increased equally with that of Portland cement type I and silica sand. Thus, the water was increased to 0.698 by the weight ratio. Table 1 summarized the mixture composition of mortar matrix in two cases.

The samples were cast in cylindrical molds with 100 mm in diameter and 200 mm in height. The silica sand, cement, and fly ash were dry mixed for about 2 min; then, the fibers were spread slowly into the mixer and continue fly mixed for 1 min. Finally, the mixing water was added and mixed for 2 min. The freshly mixed SMA fiber-reinforced mortar was cast into the molds and vibrated simultaneously by external tapping to remove any air remained in the mortar. After casting, the specimens were stayed at laboratory in 24 h before removing the molds. The demolded specimens were stored at room temperature of 25 °C and the humidity of 95% in 28 days before taking the compressive tests.

Depend on the shape of embedded fiber, the fiber content and heat conditions, the specimens were named as Table 2. The “CR” and “DG” indicated the embedded crimped fiber or the embedded dog-bone-shaped fiber, while the number behind these symbols indicated the fiber content. The “N” or “H” referred to the non-heating or heating treatments. For each type of specimen, three samples were prepared.

### 2.2. Instruments and Test Set-Up

**Figure 4:**
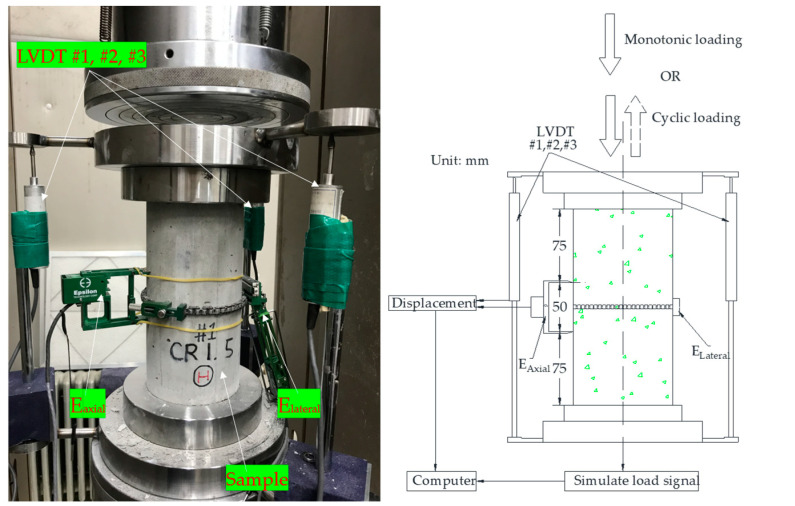
Test procedures.

The material testing system (MTS) with a 2000 kN capacity was used for the monotonic and cyclic compressive tests. To measure the deformation in the axial direction, three linear voltage displacement transducers (LVTD #1, #2, and #3) and an extensometer (E_axial_) were set up. The LVDTs created an angle of 120° around the tested specimen, and the E_axial_ with the gauged length of 50 mm was set up at the middle-height of the sample. The response deformations of three LVDTs were used as back-up for the axial extensometer, when it is failed to measure the deformation of specimen. The lateral deformation was gauged by the other extensometer (E_lateral_). The instruments and test set-up were presented in Figure 4.

**Figure 5:**
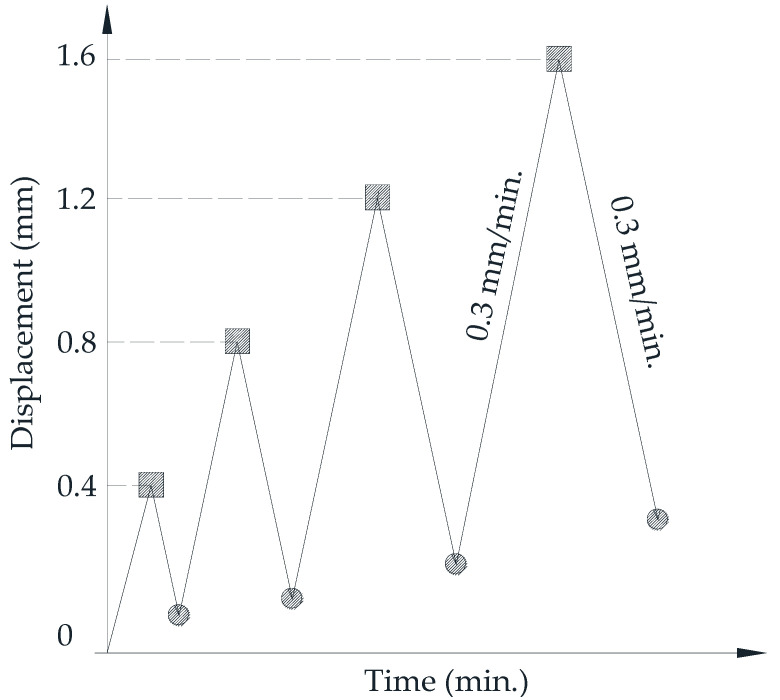
Load scheme of cyclic compressive test.

For monotonic compression, the constant load of 0.25 ± 0.05 MPa/s applied continuously and without shaking until failure. For cyclic compression, the unloading and reloading processes repeated with the step of 0.4 mm displacement and the speed of 0.3 mm/min. The starting point of reloading process at each step was set-up at the compressive force of 5 kN. The cyclic loading in a compressive test is slightly different from that in the flexural test because the reloading processes are in the same direction [22]. The load scheme of cyclic compressive test is presented in Figure 5.

## 3. Strain Behavior under Monotonic Compression

### 3.1. Axial–Lateral Strain Behavior

**Figure 6:**
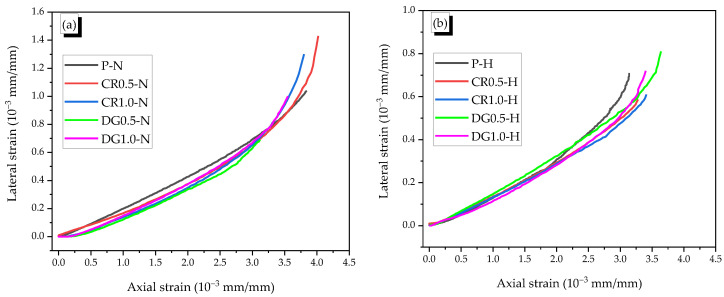
Typical axial–lateral strain curves, (**a**) Non-heating case, (**b**) Heating case.

**Figure 7:**
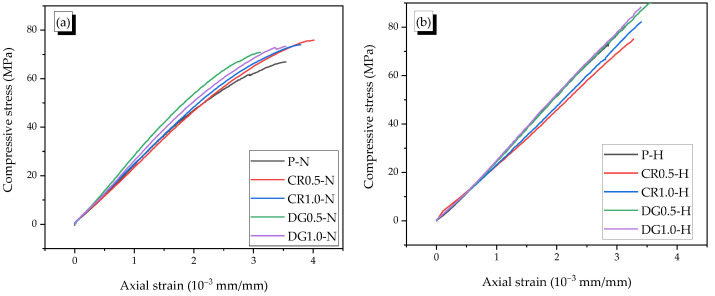
Typical axial stress–strain curves, (**a**) Non-heating case, (**b**) Heating case.

The typical axial–lateral strain curves of specimens were shown in Figure 6, and the axial stress-strain relationships were presented in Figure 7. Due to the designed mortar’s brittle property, the specimen failed just after peak stress. Thus, the ultimate strain was equal to the peak strain.

In general, when the compressive stress reached 70% mortar strength, the macrocracks started appearing and that led to the nonlinearity of the stress–strain curve. Thus, it is conjectured that the stress-strain curve was linear with the stress under 70% compressive strength. Based on ASTM C469 [23], the Poisson’s ratio was defined as the ratio of lateral strain and axial strain of specimen when the stress reached 40% mortar strength. The Poisson’s value in this part was named as $\mu_{m1}$. In the part of stress between 40% and 70% compressive strength, the microcracks formed and developed, while the effect of microcracks was insignificant. Thus, the stress-strain curve was linear. The ratio of lateral and axial strains in this part was called Poisson’s ratio $\mu_{m2}$. In this study, the axial strains at 40% and 70% compressive strength were about 1 × 10^−3^ mm/mm and 2 × 10^−3^ mm/mm, respectively. In the final part with the nonlinear relation of axial–lateral strains, the secant dilation ratio ($\beta_{m3}$) was used instead of Poisson’s ration. The $\beta_{m3}$ was defined as the slope between the first point, where the axial strain was about 2 × 10^−3^ mm/mm, and the final point of curve. The values of $\mu_{m1}$, $\mu_{m2}$, and $\beta_{m3}$ were presented in Table 3.

For the non-heating case, the Poisson’s ratios ranged between 0.15 and 0.22 in the elastic phase. Both fibers showed the effect on reducing Poisson’s ratios; in which, crimped fiber had higher influence than dog-bone-shaped fiber. Moreover, the lower fiber content was more effective than the higher fiber content in reducing the Poisson’s effect. In this case, the creation of cracks between the aggregates in the mortar matrix was significantly delayed by the presence of SMA fibers. The fibers with high tensile strength and anchorage transferred the internal tension; thus, the Poisson’s ratio of reinforced mortar became smaller than that of the plain mortar. This phenomenon was also observed with steel fiber [1,24]. The CR fiber was composited with mortar matrix better than the dog-bone-shaped fiber because the mortar teeth between the indentations of the CR fiber restricted the slip of the fiber. While DG fiber was too smooth with only two bulged diameters in the end parts, which cannot have a high composite effect with the mortar matrix. Adding SMA fibers, however, harmed the properties of reinforced mortar due to the porosity [25]. Thus, the 1.0% fiber content seemed to produce higher Poisson’s ratios compared with those of 0.5% fiber content. After cracking, there was no mortar matrix around the fiber in the opened crack; thus, the CR fiber was stretched under increasing compressive load, while the DG fiber only deformed in the elastic phase. Thus, as can be seen in Figure 6a, the lateral strain of CR fiber rapidly increased at the end part of the curve. The secant dilation ratios of specimens with CR fibers were higher by 55% and 27.5% than those of the plain and DG fibered specimens, respectively.

For the heating case, the effect of SMA fibers was unclearly in the elastic part because the Poisson’s ratios $\mu_{m1}$ and $\mu_{m2}$ of 0.13 and 0.16 for the plain specimen were not changed significantly. The specimens became harder with the heating treatment due to increasing the chemical reaction in the mortar matrix [26,27]. After cracking, there was no restriction of the mortar matrix around the fiber in cracking parts; thus, the recovery stress can recover the longitudinal deformation of the SMA fibers. This phenomenon was called the crack-closing effect, which led to the reduction of the secant dilation ratios.

The axial stress–strain curves of heated reinforced specimens all were under that of heated plain specimen (Figure 7b). The CR fiber, however, was more effective than the DG fiber for the heating case with the secant dilation ratios that were 0.22, which was below 0.3. The recovery stress of the DG fiber was higher than that of the CR fiber; for example, at the temperature of 150 °C, the values of DG fiber and the CR fiber were 317 MPa and 235 MPa, respectively. Thus, even with 1.35 times higher recovery stress, the DG fiber was less effective in reinforcing the mortar than CR fiber. The reason was relevant to the large slip of the heated DG fiber compared with that of the CR fiber in pulling out (see Figure 3).

Therefore, the SMA fiber had a positive effect of reducing Poisson’s ratio as steel fiber in non-heating. In heating, the recovery stress of SMA fiber provided the crack-closing effect for the reinforced mortar. The DG fiber was less effective in the controlled strain than CR fiber because of the larger slip.

### 3.2. Axial Stress–Strain Behavior

The effect of SMA fibers on axial strain was presented in this section through the axial stress–strain behavior of the specimens. The results of compressive strength and axial strain of specimens in the monotonic compressive test were presented in Table 4.

The data of specimen DG1.0-H#3 was not provided because a mistake occurred during the test. However, the data of samples #1 and #2 were not different, so these results can represent for DG1.0-H. The toughness ratio (TR) and elastic modulus of specimens also are shown in the last columns of the table.

**Figure 8:**
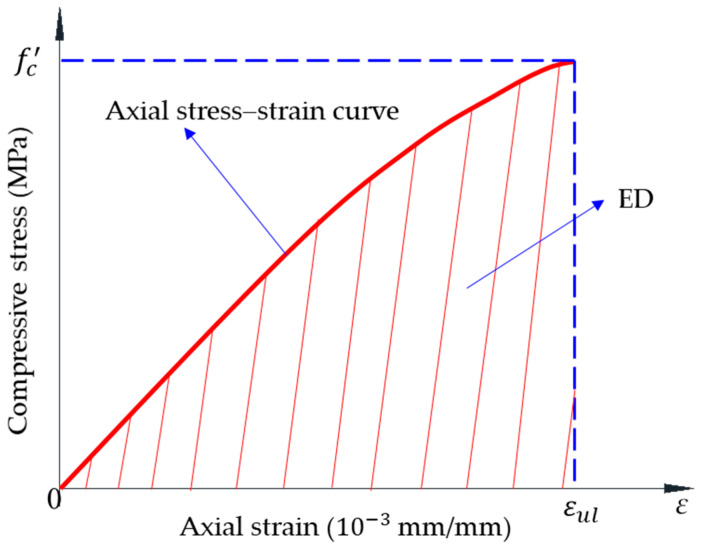
Definition of the energy absorption capacity.

The toughness ratio (TR), which was calculated by dividing the energy adsorption capacity (ED) by the multiplication of 0.015 axial strain and compressive strength ($f_{c}^{\prime })$, assessed the toughness of specimen [28,29,30]. In this study, the ultimate axial strain ($\varepsilon_{ul})$ of specimen was less than 0.015. Specimens used high ratio cement and a small size of the aggregate; thus, they failed just after reaching the peak stress. Therefore, the peak axial strain, which was the corresponding axial strain at peak stress, was equal to the ultimate axial stress. The energy absorption capacity ED was defined as the area under axial stress–strain curve of the specimen; the definition of ED was shown in Figure 8.

**Figure 9:**
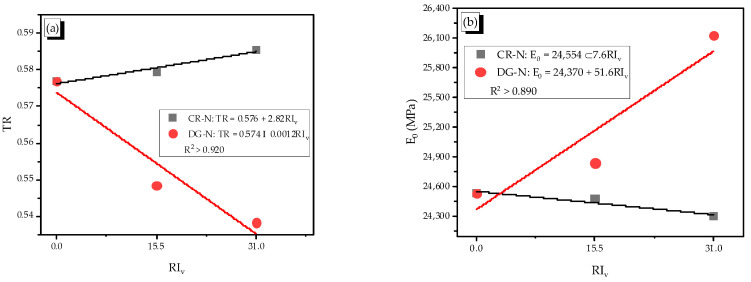
Relation of the reinforcing index–toughness ratio and elastic modulus in the non-heating case, (**a**) Relation between toughness ratio and reinforcing index in the non-heating case, (**b**) Relation between elastic modulus and reinforcing index in the non-heating case.

The reinforcing index (RI_v_), which was calculated by multiplying the volume fraction (V_f_) and ratio between fiber length (l_f_) and fiber thickness (d_f_), showed the effect of a physical parameter of SMA fibers on reinforcing behavior of the reinforced mortar. The values of reinforcing index RI_v_ were 15.5 and 31.0 with the fiber content of 0.5% and 1.0%, respectively. For the plain specimen without SMA fiber, the RI_v_ became zero value. The shape and fiber content both affected the toughness of the cementitious composite of the non-heating case (Figure 9a).

The trends of the CR and DG fibers are opposite; the CR fiber led to the linearly increasing trend while the DG fiber showed a decreasing trend. Thus, the CR fiber had higher energy dissipation capacity than the DG fiber. The indentations of crimped parts made the fiber act like a damper, which dissipated the compressive energy due to the stretching effect. While the high anchoring bond of the DG fiber made the reinforced mortar harder and reduced the toughness. The effect of the crimped SMA fiber on the toughness was similar to that of crimped or straight steel fiber [29,30]. The dog-bone shaped SMA fiber, which was only created for SMA fiber by heating the end part of straight SMA fiber, showed a different trend.

The average elastic modulus of each type of specimen was calculated as shown in Table 4. It is definitely understood that the shape of SMA fibers showed an opposite effect on toughness and the elastic modulus (Figure 9); the CR-N specimen showed the decreasing trend of the elastic modulus with adding more fiber content, while the DG-N specimen showed a reverse trend. The effect of the DG fiber was similar to the end-hooked steel fiber in a previous study [28] because they had the same pullout behavior with a high end-anchoring bond. For the CR fiber, it was stretched under a compressive load; thus, the fiber could not restrict the movement of elements in the mortar matrix. Moreover, the appearance of fibers led to the porosity in the mortar matrix; therefore, the CR fiber induced the reduction of the elastic modulus.

**Figure 10:**
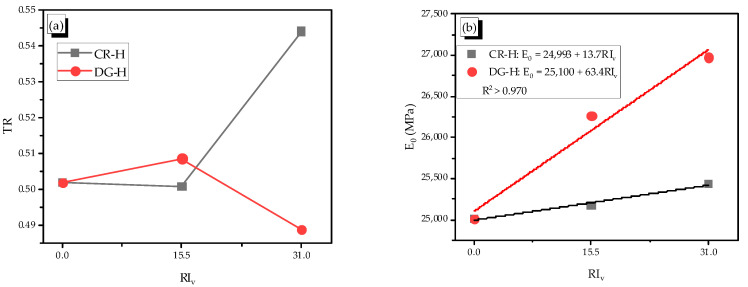
Relation of the reinforcing index–toughness ratio and elastic modulus in the heating case, (**a**) Relation between toughness ratio and reinforcing index in the heating case, (**b**) Relation between elastic modulus and reinforcing index in the heating case.

For the heating case, the heated plain specimen reduced the toughness and increased the elastic modulus compared with the non-heated plain specimen. The development of the chemical reaction due to heating was the cause of increasing adhesion between elements in the mortar matrix, which made the heated specimen harder and decreased the value of the toughness ratio [27]. The elastic modulus of reinforced mortar increased with adding more fiber content. The DG fiber was more effective than the CR fiber (Figure 10).

For the TR, it can be said that the CR fiber increased the TR overall, while the DG fiber decreased it with more adding fibers. The TRs with 0.5% are deviated from the overall trend since the fiber content of 0.5% may be not enough or the measurement might not be perfect; thus, a further studying is required for this. The elastic modulus was considered in the precracking part where the composite material worked perfectly together, while the toughness was related to pre- and post-cracking parts. After the cracking, the material worked in the plastic phase with the composite fragments connected to the others by the SMA fibers; thus, the toughness of material in this phase became complex.

### 3.3. Damage Evolution

**Figure 11:**
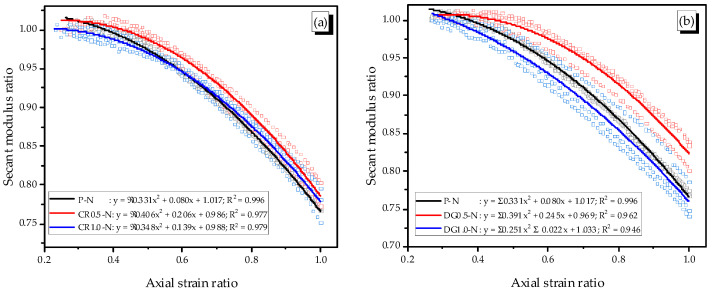
Relation of the axial strain ratio and secant modulus ratio, (**a**) P-N and CR-N specimens, (**b**) P-N and DG-N specimens.

The secant modulus ratio was defined as the ratio of secant modulus at a specific axial strain to the elastic modulus of specimen; whereas, the axial strain ratio was referred to the ratio of the specific axial strain to the corresponding axial strain at peak stress. The relationship between the two ratios is presented in Figure 11.

All specimens show a good fit with parabolic equations. As mentioned above, the stress reached 40% of the mortar peak strength at about the axial strain of 1 × 10^−3^ m/m, and then, the modulus of specimens decreased with an enlarging crack width. The corresponding axial strain at peak stress was about 2.9–4.4 (× 10^−3^ mm/mm); thus, the curves were started from about an axial strain ratio of 0.25 or 0.3 (× 10^−3^ mm/mm).

In general, the curves of the reinforced specimens with 0.5% fiber content were placed above that of the plain specimen. That indicated that the 0.5% fiber content of the SMA fiber reduced the speed of damage. At the same axial strain ratio, the secant modulus ratio of the reinforced specimens was higher than that of the plain specimen. Thus, the SMA fibers affect the reduction of secant damage after the cracking. Meanwhile, the effect of the DG fiber was higher than the CR fiber. This phenomenon was a little different in the elastic phase of specimens with the positive effect of the DG fiber and negative effect of the CR fibers on increasing the elastic modulus of the reinforced mortar. More adding fibers led to an increasing speed of damage; the curves of the 1.0% specimens were under those of the 0.5% specimens.

## 4. Strain Behavior under Cyclic Compression

### 4.1. Axial–Lateral Strain Behavior

**Figure 12:**
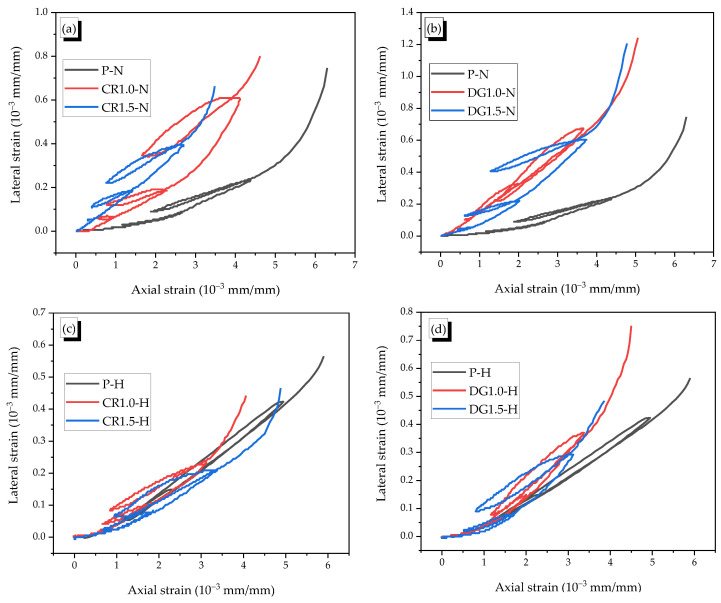
Typical axial–lateral strain curves (**a**) P-N and CR-N specimens, (**b**) P-N and DG-N specimens, (**c**) P-H and CR-H specimens, (**d**) P-H and DG-H specimens.

The typical axial–lateral strain curves of specimens under cyclic loading are presented in Figure 12. The secant dilation ratios of $\beta_{c1},\;\beta_{c2},\;\mathrm{and}\;\beta_{c3}\;$ were calculated as the ratio of lateral strain to axial strain of the specimens from the three hysteretic loops and are listed in Table 5.

In general, the SMA fibers were ineffective on controlling the lateral strain while they showed a positive effect on reducing the axial strain. The effect became significantly clear as the cyclic loading was accumulated. For the non-heating case, the SMA fibers led to a reduction in axial strain with a relative high slope in axial–lateral strain curves of the reinforced specimens compared with that of the plain specimen. The values of $\beta_{c1},\;\beta_{c2},\;\mathrm{and}\;\beta_{c3}\;$ of the reinforced specimens were approximately 2 times larger than those of the plain specimen. During the unloading process, the non-heated specimens with the SMA fibers were not effective on reducing the lateral recovery strain because the strain was too small. However, the fibers significantly restricted the recovery deformation in the axial direction. The fibers with a high anchoring bond and high tensile strength prevented the movement of two mortar matrix parts at the fibers’ end parts, and, thus, the secant dilation ratios of $\beta_{c1},\;\beta_{c2},\;\mathrm{and}\;\beta_{c3}\;$ increased. In the case of the monotonic test, there was not an unloading process. The continuous compressive load in the monotonic test created the pressure to restrict the slip between the fragments. Therefore, the loading types of monotonic or cyclic loading affected the straining behavior. The CR fiber was more effective than the DG fiber with more strain reduction of 1 and 2 (× 10^−3^ mm/mm), respectively. This phenomenon can be explained by the higher composite capacity and the higher absorbed energy capacity of the crimped shape. (Figure 12a,b).

For the heating case, the specimens showed the hardening behavior because the increment of the chemical reaction due to heating induced an enhancement of adhesion at the interface of the mortar matrix. The reinforced specimens also showed the contribution of recovery stress. The CR fiber reduced both axial and lateral strains, while the DG fiber seemed to show a relatively low effect in reducing the lateral strain. For example, the DG1.0-H specimen showed a little higher ultimate lateral strain compared with the P-H specimen. This phenomenon, which was caused by the losing anchoring bond of the DR fiber, was also observed in the monotonic test.

In conclusion, the axial–lateral strain behavior of reinforced mortar was different due to the load types. However, in both load cases, the CR fiber was more effective than DG fiber because of the high bond resistance.

### 4.2. Axial Stress–Strain Behavior

**Figure 13:**
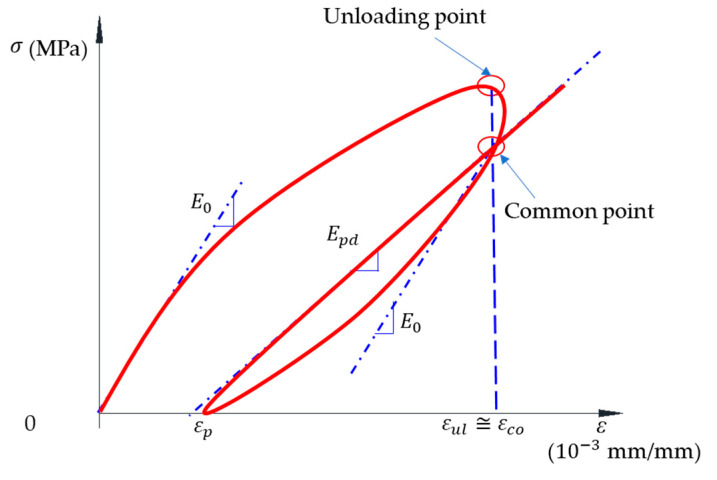
Definition of parameters in the axial stress–strain relation of the cyclic test.

The elastic modulus $\left(E_{0}\right)$ and plastic-damage modulus $\left(E_{pd}\right)$ were calculated for the cyclic compressive test. The value of the elastic modulus reduced due to damage, and the plastic strain was recorded after each cycle; thus, the slope of the reloading curves after the unloading was called the plastic-damage modulus. Figure 13 illustrates the definition of these moduli.

According to a few previous studies with steel fibers, the $E_{pd}$ was determined using the stress–strain values at the unloading and reloading points. The studies investigated the post-peak branch because, in the prepeak branch, the degradation of the modulus was insignificant and, thus, can be ignored [31].

In this study, the intersected point of reloading and unloading branches was called as a common point; the strain at that point was namely $\varepsilon_{co}$; this strain was almost equal to the unloading strain ($\varepsilon_{ul}$). The reloading branch showed the linear relation, and the slope of this line was indicated as the plastic-damage modulus $\left(E_{pd}\right)$. Each specimen experienced three cycles before failure, and the value of $E_{pd}$ for the first cycle is nearly equal to the $E_{0}$ because the loop developed at the low level of stress. Thus, the first row in Table 6 showed the plastic-damage modulus for the first cycle, and, thus, they were also the elastic modulus of the specimen.

**Figure 14:**
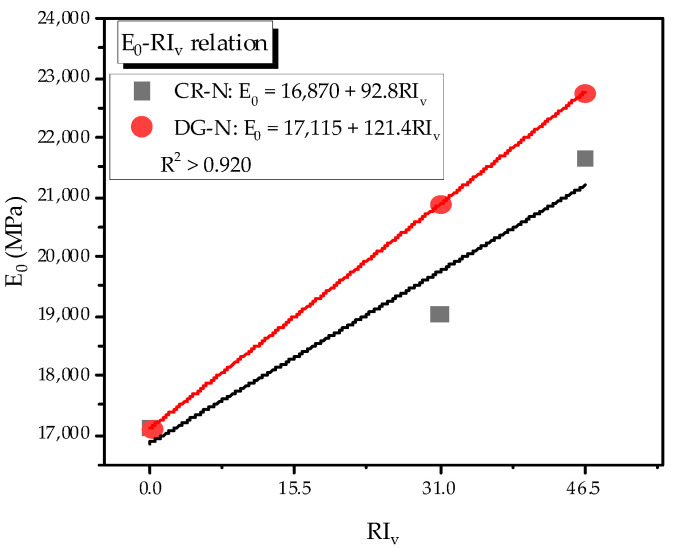
Relation of the reinforcing index and elastic modulus.

In general, the elastic modulus of mortar under cyclic loading was smaller than that with monotonic loading; which was related to the difference in mortar composition. The reinforced specimens showed relatively high elastic modulus and plastic damage modulus in comparison with the plain specimen (Table 6). For the non-heating case, the elastic modulus increased from 11 to 33% when adding SMA fibers, and the DG fiber was more effective than the CR fiber. This trend was similar to that in the monotonic test for DG fiber, while the trend of the CR fiber was different. It should be mentioned that the loading part in the first cycle was similar to that of the monotonic test. The difference may be caused by the difference in the elastic modulus of the mortar matrix and the embedded fibers [2]. The elastic moduli of the monotonic mortar and cyclic mortar were 24.5 GPa and 17.0 GPa, respectively, while that of the SMA fiber was 21.2 GPa. In the monotonic test, the elastic modulus of the mortar matrix was higher than that of SMA fibers; thus, the SMA fibers had a small effect on increasing the property of the composite material. Especially, when the fibers were stretched or slipped too much, they even showed a negative effect on the elastic modulus due to the porosity. However, in the case of the cyclic test, the higher elastic modulus of SMA fibers led to an increment in the elastic modulus of the composite material. The influence of difference between the elastic modulus of the cement-based matrix and steel fibers on the property of cementitious material was also investigated, and the same conclusion was drawn [32]. Recently, Dehghani et al. [33] also indicated that the 1.0% and 1.5% fiber content of SMA fibers increased the elastic modulus of the self-compacting cementitious composite. The mortar matrix’s elastic modulus was about 20.0 GPa, whereas the elastic moduli of the cementitious composite reinforced with 1.0% and 1.5% fiber contents were 23.0 and 21.0 GPa. The reinforcing index was proportional to the elastic modulus with both SMA fibers for the case using the mortar matrix with a relatively low elastic modulus (see Figure 14).

### 4.3. Damage Law

In the previous studies, the damage evolution law in the ascending branch was ignored because the material presented almost the linear behavior, and, moreover, it was difficult to gauge exactly the damage before the peak stress. However, the damage developed from approximately 40% of the peak stress with the initial microcracks. Then, the plastic damage modulus decreased with increasing crack width. Especially, when the microcracks enlarged to macrocracks at about 70% of the peak stress, they significantly influenced the damage law. The damage in the prepeak branch of the plain or the steel fiber-reinforced specimens were clearly observed [29,30]. The damage decrease after peak stress was indicated by the damage index, which is a function of the elastic modulus and plastic-damage modulus [31,32]. However, in this study, the specimens did not show the post-peak behavior; they were fractured just after peak stress. Thus, the damage index could not be applied in this study.

**Figure 15:**
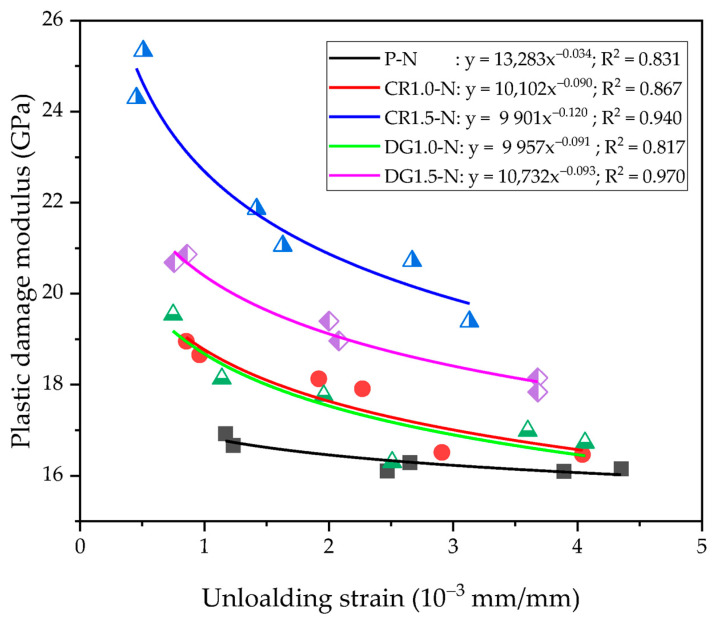
Relation of the unloading strain and plastic damage modulus.

The relationship of the unloading strain and plastic damage modulus was presented in Figure 15. The regression of the values suggested a new equation with the coefficients of *A* and *B* as below, Equation (1):

(1) $$E_{pd}=A\times \varepsilon_{ul}{}^{B}$$

**Figure 16:**
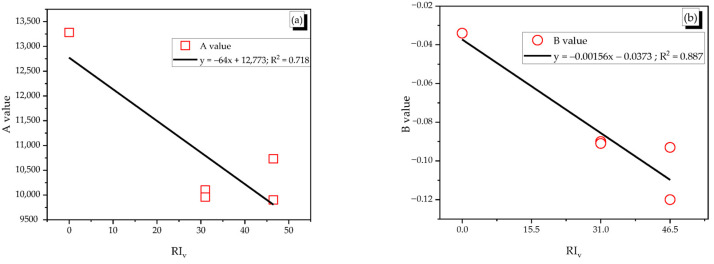
Relation between A and B coefficients and reinforcing index RI_v,_ (**a**) Relation between A coefficient and reinforcing index, (**b**) Relation between B coefficient and reinforcing index.

The value of A ranged between 9901 and 13,283, and that of B was from −0.120 to −0.034 with the correlation coefficient R^2^ over 0.817. The shape of SMA fibers was not influenced much on parameters A and B, but the fiber content changed the regressive curve. In particular, both curves of 1.0% reinforced specimens were almost matched to each other, and the curves of 1.5% reinforced specimens were placed above those of 1.0% reinforced specimens and plain specimens. Thus, this indicates that more adding SMA fibers increased the plastic damage modulus more. The reinforcing index (RI_v_) was used to indicate the effect of fiber content on the coefficients A and B. The A and B coefficients showed the linear relation with RI_v_ as see in Figure 16.

## 5. Comparison Failure Mode of Specimens under Monotonic and Cyclic Compressions

**Figure 17:**
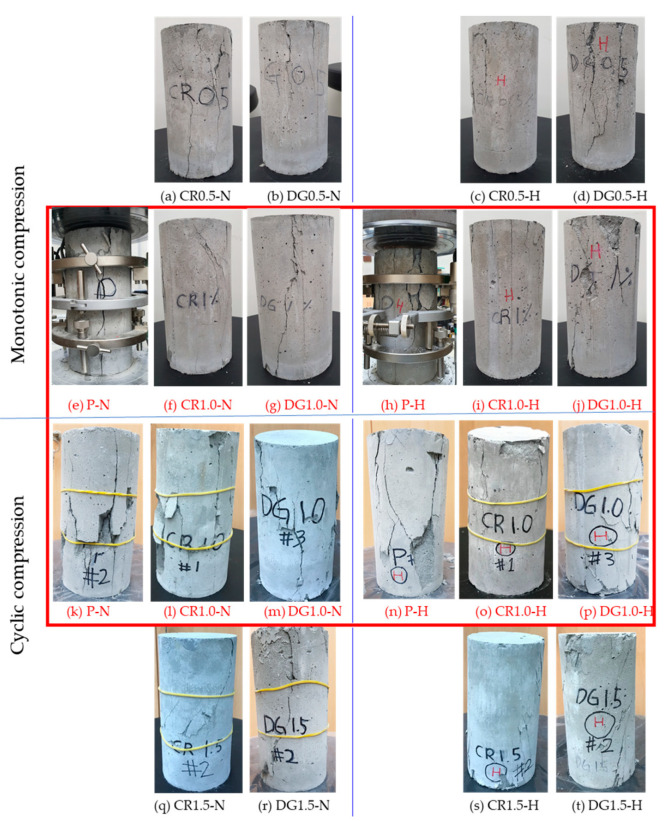
Failure of specimens under monotonic and cyclic compressive loads (**a**) CR0.5-N specimen, (b) DG0.5-N specimen, (**c**) CR0.5-H specimen, (**d**) DG0.5-H specimen, (**e**) P-N specimen, (**f**) CR1.0-N specimen, (**g**) DG0.5-N specimen, (**h**) P-H specimen, (**i**) CR1.0-H specimen, (**j**) DG1.0-H specimen, (**k**) P-N specimen in cyclic test, (**l**) CR1.0-N specimen in cyclic test, (**m**) DG1.0-N specimen in cyclic test, (**n**) P-H specimen in cyclic test, (**o**) CR1.0-H specimen in cyclic test, (**p**) DG1.0-H specimen in cyclic test, (**q**) CR1.5-N specimen in cyclic test, (**r**) DG1.5-N specimen in cyclic test, (**s**) CR1.5-H specimen in cyclic test, (**t**) DG1.0-H specimen in cyclic test.

The crack propagations of plain specimens and reinforced specimens under two load types were shown in Figure 17. The comparison failure mode of the plain specimen and the same 1.0% reinforced specimens under two compressive types was presented in the rectangle with red color.

In general, the cracks of specimens under cyclic load were significant compared with those under a monotonic test. The specimens under cyclic load recovered deformation after each unloading process and created the space between the fragments; thus, the fragments slid in the next reloading procedure. For the monotonic test, the continuously compressive loading induced the pressure, which kept the fragments contacted together and maintained the friction between them.

The SMA fibers, moreover, presented the effect on changing the direction of principal tensile stress in the cementitious composite. The cracks of plain specimens developed in vertical while that of reinforced specimens was in the diagonal direction. The major cracks shifted to an angle of about 50–60° due to the transferring tensile stress in the mortar matrix of SMA fibers. This behavior of SMA fibers was similar to that of steel fibers in a previous study [32].

## 6. Conclusions

Based on the experiential test with two different load types, different fiber shapes and different elastic modulus of mortar matrix, the straining behaviors of the SMA fibers-reinforced specimens were investigated. Some conclusions were presented as below:

- The SMA fibers with a high tensile strength and anchoring bond helped to reduce the Poisson’s ratio of specimen. The crimped fiber was more effective than the dog-bone-shaped fiber due to the higher composite capacity. However, after cracking, the dog-bone-shaped fiber had a higher controlling strain due to the high anchoring bond while crimped fiber was uninfluenced due to the stretching effect.
- For heating, the fibers did not influence much on straining behavior in the elastic phase. In the plastic phase, the dog-bone-shaped fiber slips larger than the crimped fiber; thus, crimped fiber had a higher effect on reducing strain in post-cracking.
- The straining behavior was different with different load types. For the cyclic test, the non-heated SMA fibers reduced the axial recovery deformation significantly in the unloading process; thus, the axial strain of reinforced specimens was less than that of the plain specimen.
- The SMA fibers were effective increasing the elastic modulus of the composite mortar when adding into the lower elastic modulus matrix; however, they did not influence much in the higher elastic modulus matrix or even had a negative effect due to the porosity.
- The speed of damage in the monotonic test was slow when adding a 0.5% volume fraction of SMA fibers; however, the higher fiber content did not have any effect. For the cyclic test, the damage law was presented by an equation, which depended on the fiber content, length, and thickness of SMA fibers.

## Figures and Tables

**Table 1:** Mixture composition of mortar matrix with the weight ratio.

Type of Test	Portland Cement Type I	Silica Sand	Fly Ash	Water	Water Reducing Admixture
Monotonic compression	1.0	1.0	0.15	0.35	0.009
Cyclic compression	1.0	1.0	1.0	0.698	0.0

**Table 2:** Matrix of mortar reinforced fiber cylinder.

Type of Test	Specimen	Crimped Fiber			Dog-Bone-Shaped Fiber			Heating	Sample
		0.5%	1.0%	1.5%	0.5%	1.0%	1.5%		
Monotonic compression	P-N								3
	CR0.5-N	√							3
	CR1.0-N		√						3
	DG0.5-N				√				3
	DG1.0-N					√			3
	P-H							√	3
	CR0.5-H	√						√	3
	CR1.0-H		√					√	3
	DG0.5-H				√			√	3
	DG1.0-H					√		√	3
Cyclic compression	P-N								3
	CR1.0-N		√						3
	CR1.5-N			√					3
	DG1.0-N					√			3
	DG1.5-N						√		3
	P-H							√	3
	CR1.0-H		√					√	3
	CR1.5-H			√				√	3
	DG1.0-H					√		√	3
	DG1.5-H						√	√	3

**Table 3:** Poisson’s ratios and secant dilation ratio in the monotonic compressive test.

Type	Poisson’s Ratio $\mu_{m1}$	Poisson’s Ratio $\mu_{m2}$	Secant Dilation Ratio $\beta_{m3}$
P-N	0.21	0.22	0.33
CR0.5-N	0.15	0.20	0.51
CR1.0-N	0.17	0.21	0.51
DG0.5-N	0.19	0.20	0.32
DG1.0-N	0.19	0.22	0.40
P-H	0.13	0.16	0.34
CR0.5-H	0.13	0.16	0.22
CR1.0-H	0.13	0.16	0.22
DG0.5-H	0.16	0.18	0.29
DG1.0-H	0.11	0.16	0.31

**Table 4:** Monotonic compressive properties of the specimens.

Sample	Compressive Strength (MPa)	Axial Peak Strain (10^−3^ mm/mm)	Energy Absorption	Average Strength (MPa)	Average Axial Peak Strain (10^−3^ mm/mm)	Average TR	Elastic Modulus (GPa)	Average Elastic Modulus (GPa)
P-N#1	63.0	3.6	0.141	68.0	3.7	0.58	23.04	24.50
P-N#2	74.1	3.8	0.164				24.52	
P-N#3	66.9	3.5	0.126				26.04	
CR0.5-N#1	76.4	3.9	0.165	75.5	3.8	0.58	25.50	24.48
CR0.5-N#2	75.7	4.0	0.174				23.45	
CR0.5-N#3	74.5	3.5	0.157				24.48	
CR1.0-N#1	73.4	3.7	0.163	75.0	3.9	0.59	24.92	24.30
CR1.0-N#2	75.0	3.7	0.163				26.14	
CR1.0-N#3	76.6	4.4	0.192				21.83	
DG0.5-N#1	74.7	3.2	0.126	72.5	3.4	0.55	27.65	24.84
DG0.5-N#2	71.9	3.5	0.152				23.44	
DG0.5-N#3	70.8	3.4	0.124				23.44	
DG1.0-N#1	78.1	3.8	0.173	75.7	3.7	0.54	26.44	26.13
DG1.0-N#2	73.2	3.7	0.151				25.91	
DG1.0-N#3	75.9	3.6	0.125				26.05	
P-H#1	77.0	3.2	0.126	76.1	2.9	0.50	24.41	25.01
P-H#2	71.2	2.6	0.090				25.32	
P-H#3	80.2	2.8	0.111				25.29	
CR0.5-H#1	77.9	2.9	0.108	76.9	3.0	0.50	24.89	25.18
CR0.5-H#2	77.9	2.9	0.117				25.08	
CR0.5-H#3	75.1	3.3	0.124				25.56	
CR1.0-H#1	91.9	3.3	0.174	84.7	3.2	0.54	27.57	25.43
CR1.0-H#2	80.4	3.2	0.132				25.19	
CR1.0-H#3	82.0	3.2	0.138				23.54	
DG0.5-H#1	77.6	3.5	0.137	80.9	3.4	0.51	24.05	26.27
DG0.5-H#2	83.3	3.2	0.138				28.48	
DG0.5-H#3	81.6	3.3	0.139				26.27	
DG1.0-H#1	88.4	3.4	0.149	86.4	3.2	0.49	26.97	26.97
DG1.0-H#2	84.4	3.0	0.123				26.97	

**Table 5:** Secant dilation ratio after each cycle in the cyclic compressive test.

Type	Average Secant Dilation Ratio $\beta_{c1}$	Average Secant Dilation Ratio $\beta_{c2}$	Average Secant Dilation Ratio $\beta_{c3}$
P-N	0.04	0.08	0.27
CR1.0-N	0.09	0.22	0.40
CR1.5-N	0.13	0.16	0.34
DG1.0-N	0.17	0.20	0.37
DG1.5-N	0.13	0.22	0.57
P-H	0.08	0.10	0.14
CR1.0-H	0.07	0.09	0.20
CR1.5-H	0.06	0.09	0.17
DG1.0-H	0.09	0.15	0.30
DG1.5-H	0.07	0.15	0.26

**Table 6:** Average modulus after each cycle of specimen.

**Cycle**	**P-N**		**CR1.0-N**		**CR1.5-N**		**DG1.0-N**		**DG1.5-N**	
	$\varepsilon_{ul}=\varepsilon_{co}$	$E_{pd}$	$\varepsilon_{ul}=\varepsilon_{co}$	$E_{pd}$	$\varepsilon_{ul}=\varepsilon_{co}$	$E_{pd}$	$\varepsilon_{ul}=\varepsilon_{co}$	$E_{pd}$	$\varepsilon_{ul}=\varepsilon_{co}$	$E_{pd}$
The first	1.25	17.11	0.95	19.02	1.64	21.67	0.76	20.89	0.64	22.76
The second	2.67	16.95	2.29	18.38	3.19	19.64	1.96	17.83	1.77	21.72
The third	4.38	16.34	4.09	16.75	4.99	17.67	3.66	17.34	3.10	20.41
**Cycle**	**P-H**		**CR1.0-H**		**CR1.5-H**		**DG1.0-H**		**DG1.5-H**	
	$\varepsilon_{ul}=\varepsilon_{co}$	$E_{pd}$	$\varepsilon_{ul}=\varepsilon_{co}$	$E_{pd}$	$\varepsilon_{ul}=\varepsilon_{co}$	$E_{pd}$	$\varepsilon_{ul}=\varepsilon_{co}$	$E_{pd}$	$\varepsilon_{ul}=\varepsilon_{co}$	$E_{pd}$
The first	1.63	12.55	0.74	18.93	0.69	19.49	0.91	21.35	0.62	20.71
The second	3.45	12.43	1.84	17.63	1.86	19.44	2.00	21.33	1.72	19.21
The third	-	-	3.10	17.61	3.40	18.25	3.38	20.50	3.11	18.16

* Unit of $\varepsilon_{int}$, $E_{pd}$ were 10^−3^ mm/mm and GPa, respectively.

## References

[1] Singh H. (2016). Steel Fiber Reinforced Concrete: Behavior, Modelling and Design.

[2] Zheng Z., Feldman D. (1995). Synthetic fibre-reinforced concrete. Prog. Polym. Sci..

[3] Poon C.-K., Zhou L.-M., Jin W., Shi S.-Q. (2005). Interfacial debond of shape memory alloy composites. Smart Mater. Struct..

[4] Kim D.J., Kim H.A., Chung Y.-S., Choi E. (2014). Pullout resistance of deformed shape memory alloy fibers embedded in cement mortar. J. Intell. Mater. Syst. Struct..

[5] Choi E., Kim D., Chung Y.-S., Nam T.-H. (2014). Bond–slip characteristics of SMA reinforcing fibers obtained by pull-out tests. Mater. Res. Bull..

[6] Kim M.K., Kim D.J., Chung Y.-S., Choi E. (2016). Direct tensile behavior of shape-memory-alloy fiber-reinforced cement composites. Constr. Build. Mater..

[7] Farmani M.A., Ghassemieh M. (2016). Shape memory alloy-based moment connections with superior self-centering properties. Smart Mater. Struct..

[8] Kim M.K., Kim D.J., Chung Y.-S., Choi E. (2019). Effects of a Short Heat Treatment Period on the Pullout Resistance of Shape Memory Alloy Fibers in Mortar. Materials.

[9] Choi E., Mohammadzadeh B., Hwang J.-H., Kim W.J. (2018). Pullout behavior of superelastic SMA fibers with various end-shapes embedded in cement mortar. Constr. Build. Mater..

[10] Choi E., Kim H.S., Nam T.-H. (2020). Effect of crimped SMA fiber geometry on recovery stress and pullout resistance. Compos. Struct..

[11] Choi E., Ho H.V., Jeon J.-S. (2020). Active Reinforcing Fiber of Cementitious Materials Using Crimped NiTi SMA Fiber for Crack-Bridging and Pullout Resistance. Materials.

[12] Choi E., Ostadrahimi A., Park J. (2020). On mechanical properties of NiTi SMA wires prestrained by cold rolling. Smart Mater. Struct..

[13] Choi E., Kim D.J., Chung Y.-S., Kim H.S., Jung C. (2014). Crack-closing of cement mortar beams using NiTi cold-drawn SMA short fibers. Smart Mater. Struct..

[14] Lee K.-J., Lee J.-H., Jung C.-Y., Choi E. (2018). Crack-closing performance of NiTi and NiTiNb fibers in cement mortar beams using shape memory effects. Compos. Struct..

[15] Lee J.-H., Lee K.-J., Choi E. (2018). Flexural capacity and crack-closing performance of NiTi and NiTiNb shape-memory alloy fibers randomly distributed in mortar beams. Compos. Part B Eng..

[16] Choi E., Nam T.-H., Cho S.-C., Chung Y.-S., Park T. (2008). The behavior of concrete cylinders confined by shape memory alloy wires. Smart Mater. Struct..

[17] Choi E., Hong H.-K., Kim H.S., Chung Y.-S. (2013). Hysteretic behavior of NiTi and NiTiNb SMA wires under recovery or pre-stressing stress. J. Alloy. Compd..

[18] Choi E., Kim D.J., Youn H., Nam T.-H. (2015). Repairing cracks developed in mortar beams reinforced by cold-drawn NiTi or NiTiNb SMA fibers. Smart Mater. Struct..

[19] Choi E., Mohammadzadeh B., Kim D., Jeon J.-S. (2018). A new experimental investigation into the effects of reinforcing mortar beams with superelastic SMA fibers on controlling and closing cracks. Compos. Part B Eng..

[20] Sherif M.M., Khakimova E.M., Tanks J., Ozbulut O.E. (2018). Cyclic flexural behavior of hybrid SMA/steel fiber reinforced concrete analyzed by optical and acoustic techniques. Compos. Struct..

[21] Choi E., Mohammadzadeh B., Hwang J.H., Lee J.H. (2019). Displacement-recovery-capacity of superelastic SMA fibers reinforced cementitious materials. Smart Struct. Syst..

[22] Kytinou V.K., Chalioris C.E., Karayannis C.G., Elenas A. (2020). Effect of steel fibers on the hysteretic performance of concrete beams with steel reinforcement—Tests and analysis. Materials.

[23] ASTM (2014). ASTM C469/C469M-14: Standard Test Method for Static Modulus of Elasticity and Poisson’s Ratio of Concrete in Compression.

[24] Mangat P., Azari M.M. (1985). Influence of steel fibre and stirrup reinforcement on the properties of concrete in compression members. Int. J. Cem. Compos. Light. Concr..

[25] Dehghani A., Aslani F. (2020). The synergistic effects of shape memory alloy, steel, and carbon fibres with polyvinyl alcohol fibres in hybrid strain-hardening cementitious composites. Constr. Build. Mater..

[26] Saad M., Abo-El-Enein S., Hanna G., Kotkata M. (1996). Effect of temperature on physical and mechanical properties of concrete containing silica fume. Cem. Concr. Res..

[27] Ma Q., Guo R., Zhao Z., Lin Z., He K. (2015). Mechanical properties of concrete at high temperature—A review. Constr. Build. Mater..

[28] Ezeldin A.S., Balaguru P.N. (1992). Normal-and high-strength fiber-reinforced concrete under compression. J. Mater. Civ. Eng..

[29] Nataraja M., Dhang N., Gupta A. (1999). Stress–strain curves for steel-fiber reinforced concrete under compression. Cem. Concr. Compos..

[30] Bhargava P., Sharma U.K., Kaushik S.K. (2006). Compressive Stress-Strain Behavior of Small Scale Steel Fibre Reinforced High Strength Concrete Cylinders. J. Adv. Concr. Technol..

[31] Krahl P.A., Gidrão G.D.M.S., Carrazedo R. (2019). Cyclic behavior of UHPFRC under compression. Cem. Concr. Compos..

[32] Li B., Xu L., Chi Y., Huang B., Li C. (2017). Experimental investigation on the stress-strain behavior of steel fiber reinforced concrete subjected to uniaxial cyclic compression. Constr. Build. Mater..

[33] Dehghani A., Aslani F. (2020). The effect of shape memory alloy, steel, and carbon fibres on fresh, mechanical, and electrical properties of self-compacting cementitious composites. Cem. Concr. Compos..

